# Motivation to Consent and Adhere to the FORT Randomized Controlled Trial

**DOI:** 10.3390/curroncol29040232

**Published:** 2022-04-17

**Authors:** Christine Maheu, Valerie Lok, Jacqueline Galica, Mali Tse, Emma Maltus, Lauriane Giguère, Wing Lam Tock, Sophie Lebel

**Affiliations:** 1 Ingram School of Nursing, McGill University, Montreal, QC H3A 0G4, Canada; wing.tock@mail.mcgill.ca; 2 Jewish General Hospital, Montreal, QC H3T 1E2, Canada; valerie.lok.ccomtl@ssss.gouv.qc.ca; 3 School of Nursing, Queen’s University, Kingston, ON K7L 3N6, Canada; jacqueline.galica@queensu.ca; 4 School of Nursing, McMaster University, Hamilton, ON L8S 4L8, Canada; tsem@mcmaster.ca; 5 Faculty of Health Sciences, University of Ottawa, Ottawa, ON K1N 6N5, Canada; emaltus@uottawa.ca; 6 Faculty of Social Sciences, University of Ottawa, Ottawa, ON K1N 6N5, Canada; lgigu023@uottawa.ca; 7 School of Psychology, University of Ottawa, Ottawa, ON K1N 6N5, Canada; slebel@uottawa.ca

**Keywords:** breast and gynecological cancer, clinical trial, cognitive-existential approach, fear of cancer recurrence, group intervention, interpretive description

## Abstract

The aim of this qualitative study was to identify the motivational factors that influence cancer survivors to participate and adhere to the fear of cancer recurrence (FCR) FORT randomized controlled trial (RCT). Fifteen women diagnosed with breast and gynecological cancer who took part in the FORT RCT were interviewed about their experience to consent and adhere to the trial. The transcribed interviews were content analyzed within a relational autonomy framework. The analysis revealed that the participants’ motivation to consent and adhere to the FORT RCT was structured around thirteen subthemes grouped into four overarching themes: (1) Personal Influential Factors; (2) Societal Motivations; (3) Structural Influences; and (4) Gains in Emotional Support. The unique structures of the trial such as the group format, the friendships formed with other participants in their group and with the group leaders, and the right timing of the trial within their cancer survivorship trajectory all contributed to their motivation to consent and adhere to the FORT RCT. While their initial motivation to participate was mostly altruistic, it was their personal gains obtained over the course of the trial that contributed to their adherence. Potential gains in emotional and social support from psycho-oncology trials should be capitalized when approaching future participants as a mean to improve on motivations to consent and adhere.

## 1. Introduction

Why do patients enter randomized controlled trials (RCTs) if they understand clearly that the trial, being under investigation, may or may not produce a treatment effect? In addition, if they are randomized to the control group, they are not likely going to experience any physical or emotional gain claimed by the tested clinical trial. RCTs that offer patients the possibility of a probable efficacious treatment to a condition they are affected and that no standard of care can provide for are likely to motivate their decisions to participate and adhere to RCTs. 

Fear of cancer recurrence (FCR), defined as the “fear, worry, or concern relating to the possibility that cancer will come back or progress” [1], is one of the unmet clinical needs that is not currently adequately addressed and not routinely provided for in cancer survivorship care. FCR manifests itself on a continuum with an estimated 49% of cancer survivors reporting moderate to severe levels of FCR [2], often referred to as clinical FCR [3]. Despite its high prevalence, FCR remains the number one unmet supportive care need, with 26% to 56% of individuals reporting receiving no support to manage their FCR [4,5]. These findings point to the need to test for effective controlled trials to assist cancer survivors to manage their FCR. Indeed, the conduct of oncology RCTs is necessary to demonstrate the efficacy and safety of novel cancer care approaches and to contribute to solid evidence that practitioners and patients can use to inform treatment decisions [6]. 

According to the published reviews of FCR psychosocial interventions trials, more FCR RCTs are needed that can produce larger treatment effects, considering that the reviewed FCR interventions revealed only small treatment effects at post-treatment [7,8,9]. Our team developed the Fear Of Recurrence Therapy (FORT), a cognitive-existential intervention that consists of six consecutive weekly sessions of 90 to 120 min offered in a closed group format and facilitated by two trained oncology health care professionals [10,11]. FORT combines the principles of cognitive-behavioral therapy (e.g., cognitive reframing and relaxation techniques) and existential therapy (e.g., redefining life priorities and confronting fears) to create a cognitive-existential (CE) approach that specifically focuses on FCR. 

The RCT version of FORT was implemented with 164 breast and gynecological cancer survivors [10,12]. While FORT was shown to be efficacious [12], the trial was not immune to the common challenges seen in RCT [13,14,15] and experienced methodological concerns with recruitment timeline, retention, and lost-to-follow-up. These methodological challenges continue to be of issue to psycho-oncology RCT, jeopardizing their ability to produce the needed evidence to improve health care interventions. 

Decisions to participate in a RCT are complex and multifaceted, often challenging a person to weigh their beliefs for and against the trial when making their decision [16]. Personal factors (e.g., altruism, perceived better care, improved personal health, personal benefits, and recommended by health care providers) are often the primary reasons for people deciding whether or not to participate in a CT [17]. Relational autonomy, that is, an individual’s self-determination such as to participate and adhere to a RCT is influenced by not only personal factors but also the social circumstances, significant relationships, political context [18,19,20], and power differences between the parties involved [21]. In the case of the FORT RCT, the parties would be reflected by the group leaders delivering the intervention and the study participants. Considering the documented low participation rate of cancer survivors in RCTs [22], the need to further understand the influential factors that play into an individual’s willingness to participate and adhere to RCTs [6] such as the motivational factors that played out in the FORT trial, there is a need for further investigation. The success of future cancer care trials depend on the proper accrual rate and adherence of the study participants, otherwise, they risk losing an opportunity to provide evidence toward a potential intervention that could improve cancer care [6]. To fill this gap, the primary research guiding this qualitative study was: What motivated breast and gynecological cancer survivors to consent and adhere to the six-week group sessions of the FORT trial for the management of FCR? A secondary aim was to explore the participants’ experience of taking part in the FORT RCT trial.

## 2. Materials and Methods

In this study, we used an interpretive descriptive methodology as developed by Thorne [23,24]. This approach was designed to explore clinical experience and how the learning could be applied to advance practice [25]. Using this approach, one first sets the research question to a clinical context in need of further inquiry. Second, the goal becomes to derive meanings from the participants’ own experience and words to arrive at answers to a clinical problem.

### 2.1. Sampling and Recruitment

Participants were recruited among those who took part in the FORT intervention and who had indicated interest in participating in an exit interview to subjectively share their experience. Participants from the FORT intervention had been recruited from three Canadian cities: Toronto, Ottawa, and Montreal. The inclusion criteria for the FORT intervention study included women who: (1) had a confirmed diagnosis of stages 0–3 breast or gynecological cancer; (2) were free from disease at the start of the study; (3) were 18 years or older; (4) had completed cancer treatment (with the exception of targeted therapy or hormonal therapy); (5) had clinical levels of FCR (as indicated by a score of 13 or greater on the severity subscale of the Fear of Cancer Recurrence Inventory [26]); (6) had clinical levels of distress (as indicated by a score of 24 or higher on the Impact of Events Scale [27,28]); and (7) were able to read and write English. All FORT participants signed a consent form to participate in the FORT intervention and the exit interview. Ethical approval was obtained from the Institutional Review Board (IRB) of each site where the FORT intervention took place (Ottawa Hospital: OHSN-REB #20140561-01H; Jewish General Hospital (JGH) #15-178; McGill University Health Centre (MUHC) #MM-CODMFLP-15-178; University Health Network (UHN)# 14-8036-CE).

From the participants who took part in the FORT RCT, two group leaders who delivered FORT (C.M. and S.L.) purposefully first identified sixteen participants among those who were assessed as having had diverse experiences and levels of engagement within the group sessions. The final sample size was determined to be until the data reached saturation. Between August 2018 and January 2020, fifteen of the sixteen participants approached agreed to take part in the current exit interview study, and the exposed data derived from this sample size were deemed reasonable and to have reached saturation, meaning that the findings and meanings were sufficiently developed.

### 2.2. Procedures

All interviews were collected in person, audio-recorded, and transcribed verbatim, which lasted between 45 and 60 min. The interviews were conducted by three of the authors (C.M., V.L., and E.M), with the last two being master-prepared nurses. They both received training and coaching in qualitative interviewing from the experienced qualitative interviewer CM. One interview was conducted in French and transcribed into English by V.L. The transcript was verified by a second bilingual researcher.

### 2.3. Instrument

An open-ended interview guide was developed by the first two and last authors to guide the open-ended interviews (supplementary file). The interview guide was first developed using the theoretical lens of relational autonomy to assist in understanding how the cancer survivors’ motivations to participate and adhere to a CT were influenced by the social and political context in which they were located. In addition to gathering interview data into the wider context of the women’s experience of taking part in the FORT RCT trial, our interview guide included questions that spoke to relational autonomy framework factors to explore what motivated participants to consider participating in a trial that had an unknown efficacy, and what motivated their adherence to remain in the trial. We specifically asked participants to speak as to how they made the decision to participate in the trial and whether they had engaged family members, health care providers, or others to make their decision to consent. We pursued the line of inquiry by asking participants to describe any benefits experienced from their participation and what influenced their adherence to the study. The interview guide was sent in advance by email to allow the participants to reflect on the questions prior to the interview date. Interviews ranged in length from 45 to 60 min. To preserve the participants’ anonymity, all were identified by a code number.

### 2.4. Data Analysis Process

**Figure 1:**
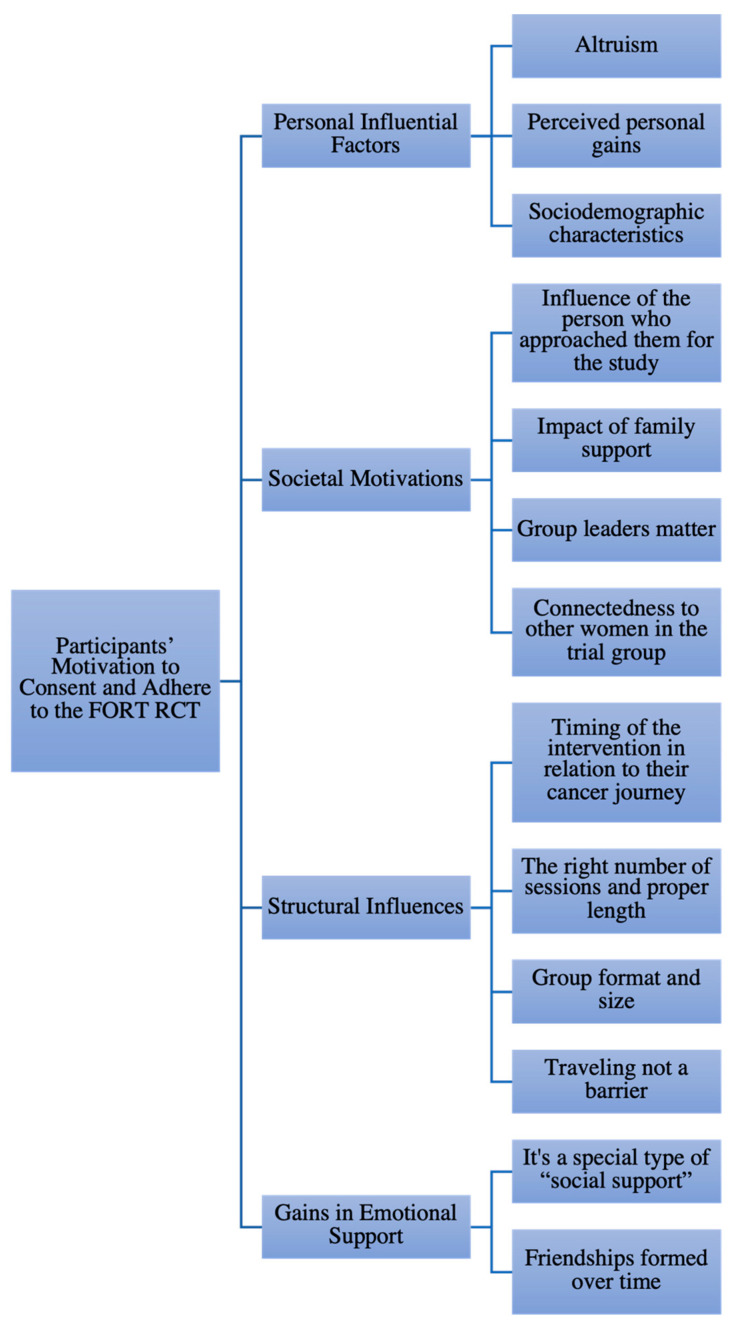
Motivators to consent and adhere: Emerging themes and subthemes.

Data collection and analysis occurred concurrently. The first and second authors analyzed the data from the open-ended interviews using the interpretive description approach [23]. Together, they first read through the transcript; on the second read, it was read through line by line, identifying specific segments that spoke to the study research questions, and identifying these through a code in which the participants’ own wording was used as the initial code. All the codes from the first six interviews were aligned and the two first authors assessed how some of the codes could be condensed into a combined thematic code. The creation of thematic coding involved grouping codes that were related or shared similar characteristics. The grouping of codes into thematic coding led to a provisional coding framework with four tentative grouping factorial themes that reflected the participants’ experience in participating and adhering to the FORT’s RCT. The preliminary coding framework was shared with the research team, along with the coded interviews to obtain their review and agreement to the themes formulated from the combined coding. This step resulted in a reconfiguration of the themes and coding framework. Following an additional six interviews, the first two authors repeated this process with the research team, which led to further refinement of the coding framework with subsequent thirteen subthemes (Figure 1). The framework was then used to code the three remaining interviews. By the end of all fifteen interviews, the team was confident that there were sufficient commonalities in the data to conceptualize meaningful and applicable findings that accurately reflected the participant’s motivation to consent and adhere to the FORT trial while providing a general description of their experience in taking part in this trial.

## 3. Results

The fifteen participants’ mean age was 53.3 (SD = 12, range = 36–76). The majority had a university or college degree (93.4%) and most had an income above $40 000 (78.6%). Seven of the participants had gynecological cancer and eight had breast cancer. Civil status was married for the majority (53.3%). A full description of the participant characteristics is found in Table 1.

As shown in Figure 1, the analysis revealed that the participants’ motivation to consent and adhere to the FORT RCT was structured around thirteen subthemes grouped into four overarching themes: (1) Personal Influential Factors; (2) Societal Motivations; (3) Structural Influences; and (4) Gains in Emotional Support.

### 3.1. Theme 1: Personal Influential Factors

Personal influential factors had a huge impact on the initial commitment of each participant to consent and adhere to the six-week group sessions of the FORT trial. In their interviews, all of the participants alluded to ideas of altruism and perceived personal gains as central motivators to their participation. Even before the start of the study, they viewed the group format as an opportunity to help others and perhaps be helped in return in the management of FCR.

#### 3.1.1. Altruism

Walsh and Sheridan’s [17] definition of altruism was used to define this subtheme: “the act of unselfishness for contribution to medicine and science” (p. 28). Each of the participants interviewed described a selfless desire to help other women who experienced clinical levels of FCR: “*I feel strong enough that I can do it if I can help the study and help other women who are in that position then I think it’s a positive thing and that’s what I want to participate in*” (P2). Two of the participants were health care providers and described their understanding of the need to improve care through evidence-based research. Others (P1, P3, P6, and P10) believed in the positive impact of clinical research. One participant (P3) described: “*I just feel like it’s the least I can do. You know, for the medical community*”.

#### 3.1.2. Perceived Personal Gains

Every participant believed that participating in the FORT trial would result in personal gains such as obtaining social and emotional support, in addition to receiving help to manage their FCR. Sometimes, these personal gains were perceived after a process of weighing the pros and cons of participating in the FORT trial where they judged the personal gains to be greater than the drawbacks. The gains expressed were wanting to learn to get “*over the fear (FCR), and learn to control it better*” (P14). Another participant (P1) stated how “*I can’t understand how it would do anyone any harm. [Especially if] we really need it*”. Two other participants (P1 and P5) believed the FORT intervention did not have any risks. As they described, they knew that they were free to drop out of the group at any time and understood that they had no obligations to participate in all six sessions of FORT. They clearly articulated their understanding that they were participating in a trial and the group support was not part of their usual care. As expressed by P4, “*the offer to take part in a group trial comes at a good time when at the end of our treatment, we are left to feel like there is no one left to help—and just the follow up from the doctor is not enough.*” Another participant (P10) explained how she did not feel that she gained anything from the FORT intervention. She explained that, following the end of the trial, she did not feel she had learned ways to better manage her FCR anxiety and did not feel that the group provided her with vicarious learning that she did not already have. However, she did recognize that the social connection and support obtained from the group were of value to her. The majority, however, expressed how “*they got something they did not realize they would*” (P2) from their participation in the trial.

#### 3.1.3. Sociodemographic Characteristics

Some participants described how personal characteristics such as age, gender, and religion influenced their decision to participate in the trial. Two women described having more time to participate in the FORT intervention because they did not have young children at home (P2) or were not employed (P4) and so had more liberty in their schedule to attend the sessions. In addition, every participant’s account alluded to the importance of having a “women-only” group because of the sharing of intimate personal details, the connection found through similar cancer experiences, and the friendships developed after the support group ended. One participant (P5) explained how her religion and faith, which she described as “*a positive way at looking at and appreciating life*”, were crucial elements in her decision to help herself and take part in the trial. Her accounts illustrated how having faith gave her strength, allowed her to believe in its importance, and helped her give up some of her own control during the group intervention. These elements helped her get through some of the more existential group exercises during the sessions such as building a worst-case scenario [29].

### 3.2. Theme 2: Societal Motivations

This theme is closely related to the concept of significant relationships in the relational autonomy framework. The subthemes that emerged from the participants’ accounts of their motivations to participate in the trial were often contextualized within the influence of their surroundings and social circles.

#### 3.2.1. Influence of the Person Who Approached Them for the Study

As part of their interview, participants were asked about who first approached them to participate in the FORT trial, and how they felt about being approached for such a study. Four participants (P1, P2, P5, and P6) described being approached for the study by their oncology nurse and identified how they had trust in this person. The participants trusted that if their nurse had recommended the study, it was because they could benefit from participating in the research study. The request to participate and enroll in the trial was most times made by a member of their health care team, someone with whom they identified as having trust in. As P2 noted, “*she was my nurse navigator (laughter)…I was very close with her already. If she thought it would be good for me to participate, I did not second guess that*”. However, even when participants mentioned that they came across the study through a pamphlet placed in the cancer community center they attended (P9), or seeing a poster in their hospital waiting room (P12), or approached by their oncologist (P13), they still felt compelled to participate as the trial tendered to a need they had of how to manage FCR.

#### 3.2.2. Impact of Family Support

The majority of the participants such as P2, P3, and P5 said that they had discussed their decision to take part in the FORT trial with family members. However, they also said that they had already made the decision to participate in the trial before bringing this topic up with their family members. Others said they had made the decision to participate independently (P1, P4, P6, P10, P12, and P13) and did not discuss their decision to participate with family members. One participant (P1), who came to her decision alone, described her independence in her decision-making: “*I didn’t feel I needed to ask anybody. I wanted to do it. And, I couldn’t see any downside*”. However, whether or not family members played a role in their decision to enroll in the trial, many had family members who were supportive of their decision: “*they were just happy for anything that I would do that would make me feel better*” (P1). However, two participants (P3 and P5) described how their husbands feared that talking about FCR would make it worse. Whereas, in reality, the participants said that being in a group of women similarly experiencing FCR made talking about it more comfortable and was helpful.

#### 3.2.3. The Group Leaders Matter

Eight health care professionals delivered FORT and the trial was available in three cities from two Canadian provinces. Four participants (P2, P3–P5, P9) specifically spoke about the same health care professional and how supported they felt in having had that person and the therapeutic relationship they felt they had developed with that specific person. Participants also alluded to the skillfulness exhibited by the group leaders in building group cohesiveness and providing a safe environment for each to have an opportunity to speak. One participant expressed it as (P2): “*I mean you [naming a group leader] were very good in terms of making sure that everybody had the chance to speak, and that we felt safe to share*”. The participants seemed to describe what would seem to be group leadership skills and competencies such as sensitivity, fairness, truthfulness, and respect to allow for authenticity and openness by the group during the sessions. A participant (P9) explained how the group facilitator’s “*approachability and compassion*” was an adhering factor to her and helped her make the decision to adhere and complete the six group sessions. 

#### 3.2.4. Connectedness to Other Women in the Trial Group

The group format in the FORT trial was that there were specific groups held for breast cancer survivors only and others for just gynecological cancer survivors. Having the group format exclusive to the type of cancer they had, participants expressed gratitude for their decision to take part in a support group with women diagnosed with the same type of cancer. The participants expressed a feeling of relatedness to the other individuals in the group. During the interviews, five participants (P2–P5, P11) were very much convinced that their mutual understanding of their cancer journey (e.g., surgery, treatment, follow-up care, etc.) contributed to their connectedness to the group and to the trial usefulness, along with the group activities that were held during those sessions, with special mention to the creation of the worst-case-scenario activity. It was P12’s view that seeing how helpful each of the weekly sessions were to herself and others in the group provided the motivation to continue attending the other sessions. These experiences of connectedness to the group and the trial likely contributed to their sense of resilience toward managing their FCR, as expressed by P13 “*I believe it [their trial participation] made me stronger, more resilient*”. Another participant (P11) stated that it was simply “*nice to be around other people with cancer*”.

### 3.3. Theme 3: Structural Influences

This theme aligns with the structural influences identified within the relational autonomy framework. The participants spoke to structural influences in the nature of the physical, social, economic, and political environment that influenced their decision to participate and remain in the trial.

#### 3.3.1. Timing of the Intervention in Relation to Their Cancer Journey

The time elapsed between the end of the participants’ cancer treatment and the beginning of the FORT trial group varied for many, and this time frame represented an important structural influential factor. For some of the participants, they were approached to take part in the trial right after completing their cancer treatment (P1), while another was approached at one of her follow-up care for cancer and was eight years into her cancer survivorship phase (P2). Both participants had the same type of cancer and both had clinical levels of FCR. However, it was the specific time difference between the end of their cancer treatment and the start of the support group that seemed to play an influential role in their motivation to consent to the trial. The participant who started the FORT trial right after completing her cancer treatment explained how she had no time to talk herself out of participating: “*It [the chemo] was just finishing... I didn’t have time to stew*” (P1). She also feared the loss of the regular check-up by her oncology team now that the treatments were finished. The timing of the trial felt like it was the natural next step in her journey. Conversely, the other participant who was eight years out of treatment did not believe that starting FORT right after treatment would have been a good idea for her. In fact, her views from her experience in attending the six sessions were that the discussions around FCR were harder for the participants who had just completed treatment “*Some of them were just fresh off chemo and stuff, I think it was harder for them*” (P2). While some participants wished the FORT trial had been offered to them sooner in their cancer trajectory (P12) and even at least mentioned during their cancer treatment, others (P10, P12, P13) suggested that perhaps it would be best for future FORT participants to be a few months past their cancer treatment to ease the comfort of speaking about FCR.

#### 3.3.2. The Right Number of Sessions and Proper Length

The time commitment to participate in the FORT trial had an impact on the participants’ decision to enroll. Four participants (P3, P5, P6, and P10) felt that the six-week commitment of two hours each was reasonable. Three other participants (P7, P9, and P11), however, believed that six sessions were not enough and that eight sessions would have been best to feel like they had some control over managing their FCR. Although it was not offered in the FORT trial, many participants (P1–P6, P7, P9, and P11) believed that a booster session later following the end of the six sessions such as around six months, was all that would have been needed to make the intervention better. The participants further explained that having a booster session would also give an opportunity to “check-in” on others as they had developed strong friendship bonds and emotional connections, and felt concerned to know how their group members were doing. Among the fifteen participants, all but four mentioned remaining in contact with past group members of the FORT trial.

#### 3.3.3. Group Format and Size

In their accounts, all the participants agreed that having at least six individuals participate in the group was an optimal group size. They also spoke of their appreciation toward how no new group members were invited following the start of the group. Participants believed that having more than six participants in a group could be unmanageable for both the participants and the group facilitators. The participants described the importance of obtaining a balance between a few participants, which would feel like individual therapy, and too many participants, which would take away from their “*air time*” (P6), as each participant was regularly encouraged to share their feelings toward FCR. Participants described how the right amount of people in the group led to their feeling comfortable to openly express and share their emotions about FCR. The connectedness that was developed was lived with a sense of “*camaraderie*” (P2), ultimately leading to the type of emotional support they came to appreciate in the weekly group session.

#### 3.3.4. Travelling Not a Barrier

The participants were asked about the influence of having to travel to attend the group on their decision to take part in the FORT trial. The participants expressed that the weekly travelling to attend the group sessions in person was not seen as a barrier. While their transportation and parking costs were reimbursed as being part of the trial study, many participants (P1, P3–P6, P10, P12, and P13) expressed that these structures did not play an influence in their motivation to consent and adhere to the trial. Coincidently, most of the participants lived close to the site where the trial group was being held. Travelling time ranged from 10 to 90 min.

### 3.4. Theme 4: Gains in Emotional Support

Two distinct interpretations were found under the theme of emotional support: social support and the friendship formed within the members of the group.

#### 3.4.1. It Is a Special Type of Social Support

On initial analysis, the support the participants described receiving from their participation in the FORT group was alluded to as a type of social, which would have initially fallen into the societal motivators theme. However, after probing for explanations, the participants described the support in the group as different from the caring and encouraging support received from their health care providers and family members. Four participants (P1, P2, P3, and P5) described the FORT group as a supportive place where having a conversation was easy because of their shared and similar struggles such as with managing FCR. They said they had no other place and no other people with whom they could have this specific discussion toward their struggle with life after cancer. They also felt that few outside their group really understood what they were going through or wanted to hear of their struggles. A source of support was also the thirty minutes of free time prior to the start of each start group session where the participants were encouraged to arrive early and chit chat over snacks and drinks. Some participants said they also used this time to complete and discuss their take-home homework “*We sat together at the hospital prior to our sessions and reviewed and completed our homework together*” (P8, P12, P13). When asked what motivated them to continue and attend all six sessions, P2 responded that “*it was definitely with being with the other women, there’s no question about that.*”

#### 3.4.2. Friendships Formed over Time

The friendships formed throughout the course of the six sessions of FORT were said to have played a role in the participants’ assiduity to the group sessions and the study. Being in a group that shared the same gender, cancer type, and similar struggle with FCR brought them comfort to openly engage and share their thoughts and emotions. In getting to know each other throughout the weeks, participants also discovered other similarities that made them reach out to specific participants outside the group work hours. For example, one shared how two of them were meeting regularly for long walks and coffee. One other shared how she engaged another group member to attend her reading club meetings. The participants described that through some of the group activities in each of the sessions, they came to share very intimate details. Details of which, they explained, they would have never shared, had it not been for the sense of comfort and the non-judgmental context that the group environment provided. The sharing of intimate details led to the development of close bonds and friendships. Participant 7 described the friendships built “*as a bonus*”, since she was not expecting to make new friends through her participation in the trial. She described joining the group simply to tell her story to people. The group created a sense of kinship and closeness among the women, as evidenced by participant 2, “*It was the sense of like friendship almost that we had at the end, we were total strangers, we don’t know our last names, we don’t know where we live; yet we shared so much in the last few weeks*” Every participant described the friendships built as one of the main positive outcomes of their participation in the FORT trial.

In summary, the themes and subthemes derived from the fifteen participants’ accounts provide insight into the motivational factors and contextual influences that played a part in their openness to take part in the FORT trial and on their adherence to the trial. The findings show that personal and social aspects contributed to the participants’ motivation to consent, while the group structure and the emotional support formed contributed to their adherence in the trial.

## 4. Discussion

Our study findings offer insight into the participants’ motivations to consent and adhere to a RCT, one that focused specifically on the management of FCR. The participant’s accounts featured four major themes—personal influential factors, societal motivators, structural influences, and gains in emotional support and expanded over thirteen subthemes (see Figure 1). The findings revealed that personal and social aspects were closely related to the participant’s motivation to enroll in the FORT trial, while the group structure and the emotional bonds formed contributed to their adherence to the weekly group sessions and the trial.

An integrative review by Bell and Balneaves [6] summarized that there were three main factors and contexts that influenced cancer patients’ decision-making as it relates to participation in a trial: the personal, the social, and systems. These same features also played a significant role in our study participants’ motivation to participate in the FORT RCT, and given the group format of this current study, our results provide new insights into participation adherence to RCT studies. Indeed, our results go beyond previous reports, showing that the emotional and psychological support that women received from others in the group had a major impact on their willingness to adhere to the full six weeks of the RCT and beyond, with the completion of the follow-up measures three and six months after the last group session. These results suggest that the creation of a safe environment within the group, allowing for intimate discussion to take place, are necessary components to favor adherence into a trial. Valuing the worth of exchange during the course of the weekly group sessions and highlighting the opportunity to contribute to each individual’s journey of managing FCR are likely to influence the motivation of future participants to enter into a trial. Special attention also needs to be given to the selected person to first approach potential participants. In this study, this person was often the participants’ nurse who sought their interest. The participants, having an entrusted faith in this person, viewed their proposal to take part in the trial as likely to be a good thing for them. The connectedness also felt with the group leaders who they met in a pre-interview prior to the start of the trial to share the group study’s expectations was also an influential factor in their consenting to participate in the trial [30,31,32]. This was similarly observed in another study that looked at the influential aspect that led cancer patients to take part in a clinical cancer trial, where the decision to participate was noted as often immediate, guided by emotions, and based on a trusting relationship with health care personnel rather than on careful reading of written trial information [33].

Women’s motivation to consent to the FORT trial was primarily driven by a mix of both personal and societal influences. In a narrative review, they found that the primary factor influencing participation in clinical controlled trials amongst patients was related to personal factors, where patients associated their participation with obtaining a form of personal gain [17]. Similarly, our findings revealed that women enrolled in the FORT trial for reasons such as expected personal benefits and remained in the trial for reasons of altruism. These findings build on Walsh and Sheridan’s [17] work, which suggests that study participants over time come to find a balance between first enrolling in trials for personal or altruistic reasons, but adhere after seeing both personal gains to their participation and the ability to contribute to others and to science. As such, the participants in our study described in detail their positive experiences in the FORT weekly group sessions, how they came to learn from each other, realized they had something to contribute, how the group facilitators helped them gain better control over their FCR, and how grateful they were to have found this trial and the other participants in the group.

Whilst the present study did not specifically focus on the decision-making process related to participating in a RCT, the results indicated that in addition to the timing of the support group, the participants’ readiness for change had an influence on their decision to take part in a trial. Their willingness to consent and participate in the trial demonstrated a readiness to pursue behavioral change. As described in the findings, the participants’ decision to consent was driven by personal influences, whether altruism or perceived personal benefits. Once in the study, the emotional support obtained through the sharing of intimate details of their struggle with FCR also speaks to the need to consider the emotional readiness of the study participants prior to enrolling in psycho-social group trials such as with FORT, being a cognitive-existential group intervention. However, emotional readiness alone was not enough for the participants to adhere to the full six weeks of the support group. Other subthemes that emerged under structural influences were the time commitment required to participate in this research study and the traveling time. A majority of women stated that the total duration and number of hours required to participate in the support group were reasonable. Walsh and Sheridan’s [17] review notes that time commitment into a trial is an important barrier to participation. They further found that travel time was the greatest barrier to participation in trials [17]. In our study, however, the participants did not identify the logistics of transport and the travel time to the group sessions as barriers or having an influence over their participation in the trial. This difference may be explained by the fact most participants coincidently lived near the location where the FORT trial took place.

Finally, our study findings revealed that the participants’ motivations to adhere to the support group were strongly linked to an emotional component. The emotional support they received from the support group’s leaders and the other participants in their group showed a clear positive impact on their adherence to the trial. There was this sense of camaraderie and continued willingness to contribute to each other’s journey in managing what they saw, a similar battle they all shared—living with FCR. Our study findings revealed that almost every woman who participated in the FORT group trial developed close bonds with each other, which during the six weeks evolved into strong friendships.

As highlighted in other studies of the valuable supplemental support obtained from group studies other than from friends, family, and health care providers [34,35,36,37], in our FORT trial, the participants described experiencing a unique sense of community, unconditional acceptance, a space to learn how to cope and obtain information about cancer and its treatment, and in the management of FCR. As identified in the findings, having a female-only group, sharing the same cancer type, likely contributed to this heightened sense of community and belonging, strengthening the emotional connection they developed toward one another when opening up about their shared experiences. These shared experiences, in turn, helped them engage and contribute to the support group’s healing environment.

## 5. Study Limitations

In considering the results of the present study, several limitations were acknowledged by the authors. First, only women who had completed the full six weeks of the support group were interviewed. This limits our understanding of the women’s adherence to the support group, since women who dropped out of the support group were not interviewed. While the influence of educational level on the motivation to participate in psycho-oncology trials is still unclear [17], it must be recognized that our study sample was mostly constituted of college and university graduates. Continued examination of the socio-demographics’ influence on the willingness of the study participant to take part in a RCT is needed to ensure that we improve the study representation and improve the study’s external validity. Second, the end-of-intervention interviews took place over one year after the completion of the support group, making the ability to recall memories and decisions challenging at times. There is a possibility of selection bias toward having only those who lived close to the center where the groups were being delivered may have opted to participate in the study. While transportation is a common barrier noted for cancer survivors’ participation in trials [38], our study participants did not identify transportation as a barrier to their participation. However, participants were reimbursed for their travel costs as part of their participation, which could further contribute to selection bias. Finally, as our social lives rely increasingly more on virtual connections, this may have implications in the connections and friendships that women form during the study. As the ability to physically connect will be lost online, the lack of physical interactions may have an impact on adherence to the trial.

## 6. Implications for Practice and Research

The results from this research study have implications for practice from both a clinical and research perspective. On a clinical level, nurses should be made aware of their influential role in motivating their patients to take part in RCT. Increased implementation success could be achieved by having clinical nurses fully understand the factors that influence study participants to consent to a trial such as being aware that trust in the health professionals approaching them to participate in trials plays a big part in their motivation to enroll in the study, and emphasizing the opportunity and their potential to help others by participating in a group trial [7]. To improve on the principle of fairness and equity of research participation, we recommend specific strategies be put in place to recruit individuals from diverse socioeconomic backgrounds starting with a review of the selected locations where the trials are delivered. A research implication is how the emotional and social support within the groups were seen as having a major impact on trial adherence. Researchers should capitalize on this feature by planning on how group cohesion can be enhanced, and seeing how this group therapeutic factor specifically, along with other known group therapeutic factors [39], can be translated for virtual sessions to promote this sense of community and commonality toward achieving a common goal.

## 7. Conclusions

The four themes and thirteen subthemes identified from the fifteen participants’ accounts provide insight into the motivational factors and contextual influences that played a part in their motivation to consent and adhere to the FORT RCT. The findings show that personal and social aspects contributed to the participants’ motivation to consent, while the group structure and the emotional support formed contributed to their adherence to the trial.

## Figures and Tables

**Table 1:** Demographic and medical characteristics of the participants (*n* = 15).

Characteristics	M (SD); Range
Age	53.3 (12.0); 36–76
Time since diagnosis (years)	2.2 (2.4)
	%
Marital status	
Single	26.7
Married/Common Law	53.3
Separated/Divorced	20.0
Ethnic background	
Caucasian	73.3
Asian	20.0
Other	6.7
Working status	
Working	53.4
Not working	46.6
Education	
Part of university/college	6.7
University/college	93.4
Graduate degree	0.0
Family Income ($)	7.1
<$20,000	
$21,000–40,000	14.3
$41,000–60,000	21.4
$61,000–80,000	28.6
$81,000–100,000	0.0
>$100,000	28.6
Cancer diagnosis	
Breast	53.3
Gynecological	46.7
Cervical	14.3
Endometrial/Uterine	14.3
Ovarian	71.4
Cancer stage	
I	7.1
II	57.1
III	35.7

## Data Availability

The data analyzed in this study are available upon request from the corresponding author.

## Supplementary Materials

The following are available online at https://www.mdpi.com/article/10.3390/curroncol29040232/s1, File S1: FORT trial Interview Guide.
