# Supplementary material for: Motivation to Consent and Adhere to the FORT Randomized Controlled Trial
Source: Curr Oncol. 2022 Apr 17;29(4):2848–63. doi: 10.3390/curroncol29040232 (PMC9025660; doi:10.3390/curroncol29040232)
Supplement: Supplementary file 1 [file curroncol-29-00232-s001.zip › curroncol-1673796-SI.pdf]

Supplementary File 1. FORT trial Interview Guide

1. What can you tell me about your experience in participating in this cancer support group study?
    - *Reformulation*: To begin, can you tell me about your general experience in participating in this study?
    - *Reformulation*: What did it feel like to participate in this study?
    - *Reformulation*: How was it like to participate?
    - *Possible follow-up question:* Have you ever participated in a study before?
    - *Prompt #1*: Can you tell me more?
    - *Prompt #2:* Can you give me an example?

2. When researchers conduct studies, one difficulty they sometimes have is in finding enough individuals to participate in their studies. To this end, can you share what
    - motivated you to participate in this study?
    - Is there a specific context that influenced your decision to take part in the study?
    - *Prompt #1*: Personal reasons
    - *Prompt #2*: Personal situations?
    - *Prompt #3:* Your work situation?
    - It can happen that people who participate in research studies do not feel like they have benefited anything, or do not feel like they have gained any reward. How do you feel regarding this?
3. As I mentioned earlier, recruitment can be a problem for some researchers. Another problem is something called "attrition", where researchers cannot get participants to stay in the study.
    - What would you say motivated you to stay in this study?
    - What influenced your decision to participate in the study and to stay for the entire 6 weeks?
    - Was traveling to the site an obstacle for you? (contextual influence)
    - Did your economic situation influence your decision to participate? *Possible prompts:* having your transportation reimbursed, did it influence your decision to participate and remain in the study? (economic influence)
    - What do you think facilitated your decision to participate in the trial, and what do you think could have been barriers to declining to participate?
    - Can you describe the benefits obtained from your participation in the study, if any?
    - What did you have to do to fit the 6 weeks sessions into your schedule?

4. Sometimes, decisions are made within a context, an example of this would be a family member that influenced you to participate, other influences would be employment, altruism, etc. Can you describe if you have any factors that led you to make this decision?
    - *Reformulation:* How has your family/spouse/parents/children influenced your decision to participate in this study? (social and familial context)
    - *Possible follow-up question:* How did your family/spouse/parents/children react when you told them you were participating in a research study?
    - *Reformulation:* Do you remember some of your thoughts and feelings as you made your decision to participate?
    - *Reformulation:* Can you describe what led to your decision to participate in this clinical study?
    - *Possible prompt:* Did you speak to other healthcare professionals about this? (interpersonal ties) or were you influenced by a healthcare professional to participate?

Before we move to more specific questions from your experience with some of the weekly sessions, is there anything else you would like to add that pertains to the context in which you selected to participate in a group study and to remain in the study until completing the 6 sessions?

5. Do you think the number and length of the sessions were adequate?
6. Did this group help you be more in control of your emotions?
   Yes. In which ways?
   No. Could you please expand?
7. What was most helpful in dealing with your fear of cancer recurrence in this study?
8. What was least helpful (in terms of activities, new skills learned) with your fear of cancer recurrence?
9. What activities did you like best?
    - i.e. Practicing cognitive reframing, wheel of life, relaxation audios,
   the worrying questionnaire, the nurse visit, and question period
10. What exercises from the group sessions did you prefer and which did you not like as much?
11. What relaxation exercise helped you the most to relax? Relaxation CD, calming self-talk, guided imagery, grounding exercise, worst-case scenario?
12. Which relaxation exercise worked least for you in helping you relax?
13. Which activities worked the least to help you gain control over your fear of recurrence?
14. How did you feel about the nurse's visit in session 3?

- It is helpful to learn the signs and symptoms of possible recurrence
- Was it stressful for you to hear?

15. Do you think it was a good idea to have her come? What did you think about the length of the support group being 6 weeks and for 2 hours? Did you find enough time, too long, could have been done in less than 6 weeks?
16. Some participants have suggested adding a reunion gathering a few months after ending the 6 sessions. What are your thoughts on this? Would this be something you would be interested in attending?
17. How did you feel about the homework assignments?
18. If you could do the group again, what would you change, and what would you improve?
19. If you could do the group again, what would you keep the same?
20. Would you recommend this group for women in similar circumstances?
21. This wraps up the questions. Do you have any additional comments or questions that would help us understand better what motivates cancer survivors to participate in a study group and to remain in the study until its completion?
